# Grave’s disease as a manifestation of immune reconstitution inflammatory syndrome in an HIV-infected child on highly active antiretroviral therapy: A case report

**DOI:** 10.1016/j.idcr.2025.e02478

**Published:** 2025-12-23

**Authors:** Asteway M. Haile, Biruk T. Mengistie, Chernet T. Mengistie, Elezer B. Zewde, Addis H. Bekele, Bezawit M. Haile

**Affiliations:** a School of Medicine, College of Health Sciences, Addis Ababa University, Addis Ababa, Ethiopia; b Department of Paediatrics and Child Health, College of Health Sciences, Addis Ababa University, Addis Ababa, Ethiopia

**Keywords:** Immune reconstitution inflammatory syndrome, Graves' disease, Antiretroviral therapy, Autoimmune thyroid disease

## Abstract

**Introduction:**

Immune reconstitution inflammatory syndrome (IRIS) can unmask autoimmune disease after antiretroviral therapy (ART), and Graves’ disease has been reported as a late autoimmune manifestation, though pediatric cases are exceptionally rare.

**Case presentation:**

A 9-year-old Ethiopian boy with vertically acquired HIV, diagnosed at age 6 during an acute illness, had presented at that time with profound immunosuppression (CD4 23 cells/mm³, HIV RNA ∼150,000 copies/mL) and was started on combination ART. He achieved sustained virologic suppression and marked immune recovery (CD4 >1800 cells/mm³). Thirty-three months after ART initiation he developed a six-month history of weight loss, palpitations, increased appetite, night sweats and progressive bilateral proptosis. Examination showed tachycardia, lid retraction, lid lag and a diffusely enlarged, soft, non-tender goitre. Laboratory testing revealed suppressed TSH and elevated free T4; thyroid ultrasound demonstrated a diffusely enlarged, hypervascular gland. Thyroid autoantibodies were not available. A clinical diagnosis of Graves’ disease in the context of IRIS was made.

**Management and outcome:**

ART was continued. He was treated with carbimazole and propranolol with close endocrine and infectious-disease follow-up. Symptoms resolved, heart rate normalized and thyroid function tests returned to the euthyroid range, allowing down-titration of carbimazole to a maintenance dose.

**Conclusion:**

This case illustrates that Graves’ hyperthyroidism may present as a late IRIS manifestation in children with profound immune recovery after ART. Early recognition, standard antithyroid therapy and continuation of ART can achieve good outcomes.

## Highlights

• Rare pediatric Graves’ disease emerged as IRIS post-ART in HIV.
• Onset occurred 33 months after ART with marked CD4 immune recovery.
• Diagnosis based on thyrotoxicosis, diffuse goiter, and ophthalmopathy.
• Effective management with carbimazole and propranolol, ART continued.
• Case underscores need to suspect autoimmune thyroid disease in HIV kids.

## Introduction

Immune reconstitution inflammatory syndrome (IRIS) refers to paradoxical inflammatory reactions that occur after initiation of antiretroviral therapy (ART) in HIV-infected patients, reflecting restored immune responses to infectious or antigenic stimuli [1], [2]. While IRIS classically involves opportunistic infections, it can also trigger autoimmune conditions, including thyroid autoimmunity [2], [3], [4]. Graves’ disease is an organ-specific autoimmune disorder in which pathogenic immunoglobulins stimulate the thyroid-stimulating hormone receptor (TSH-R), causing thyroid follicular hyperplasia and excess thyroid hormone production [5]. Graves’ disease is the leading cause of hyperthyroidism in iodine-sufficient regions, affecting approximately 3 % of women and 0.5 % of men [6]. In children, Graves’ disease is rare (incidence ∼0.1–3 per 100,000; female:male ∼5:1) but remains the most common cause of pediatric thyrotoxicosis [7], [8]. Clinically, Graves’ disease presents with suppressed serum TSH, elevated free T4 (and often free T3), diffuse goiter, and ophthalmopathy [5], [7]. Diagnosis is confirmed by thyroid-stimulating immunoglobulins or TSH-R antibody testing, thyroid ultrasound Doppler showing a hypervascular gland, and the presence of ophthalmopathy [6], [7]. First-line therapy in children is long-term antithyroid medication (usually methimazole/carbimazole for 24–36 months), often with adjunct beta-blockade for symptoms [5], [6].

In HIV-infected patients, restoration of immunity on ART can precipitate autoimmune diseases as IRIS [1].This occurs because immune reconstitution in severely immunosuppressed patients expands naïve CD4⁺ T cells and may break immune tolerance, unmasking organ-specific autoimmunity [2]. Thyroid dysfunction is frequently reported in HIV (especially hypothyroidism or subclinical abnormalities), but overt hyperthyroidism due to Graves’ disease is uncommon [4], [9]. Epidemiologic studies suggest overt thyroid disease occurs in only a small minority of treated HIV patients [4]. Graves’ disease as a manifestation of IRIS has been described in adults, typically as a late phenomenon (often >12–36 months after ART) [4], [10]. Pediatric Graves’ IRIS is exceedingly rare: by 2019, only a single case had been reported in a child [11]. Thus, although Graves’ disease in children is itself rare, its occurrence as IRIS post-ART is vanishingly uncommon [2], [11].

Given this context, we report a case of Graves’ hyperthyroidism developing 33 months after ART initiation in a child with vertically acquired HIV. We discuss the epidemiology and pathophysiology of Graves’ IRIS, its typical clinical features, diagnostic considerations, and standard pediatric management strategies for autoimmune thyroid disease. This case emphasizes the importance of recognizing Graves’ disease during the immune-reconstitution phase of HIV therapy to ensure timely diagnosis and treatment.

## Case presentation

A 9-year-old Ethiopian boy presented to our tertiary hospital with a six-month history of progressive weight loss, palpitations, increased appetite, night sweats and bilateral proptosis. He had been diagnosed with vertically acquired HIV at age six (February 2021) after a two-week illness with fever, headache, cough and weight loss; baseline testing then showed severe immunosuppression (CD4 23 cells/mm³) and a plasma HIV-1 RNA of approximately 150,000 copies/mL, and he was started on a standard first-line triple-drug ART regimen, consistent with current WHO guidelines, consisting of dolutegravir, abacavir, and lamivudine. His immunovirological response to ART is summarized in Table 1.

**Table 1:** Immunovirological and Clinical Timeline.

Time Point (Months relative to ART start)	Age (Years)	CD4 Count (cells/mm³)	HIV RNA (copies/mL)	Clinical Event
0 (Baseline)	6	23	∼150,000	HIV diagnosis; ART initiated
+ 6	∼6.5	∼150	< 1000	Early immune reconstitution
+ 23	∼8	1475	< LOD	Sustained virologic control
+ 27	∼8.3	N/A	< LOD	Onset of initial symptoms (increased appetite, mild palpitations)
+ 30	∼8.5	1870	< LOD	Preceding routine follow-up
+ 33	∼9	> 1800	< LOD	Full clinical presentation & diagnosis of Graves' disease

LOD: Limit of Detection; Detectable but significantly suppressed from baseline

The earliest symptoms, notably increased appetite and intermittent palpitations, were reported retrospectively to have begun approximately 27 months after ART initiation. These symptoms progressed gradually over the ensuing six months, with the subsequent development of more pronounced weight loss, night sweats, and visible proptosis, leading to his clinical presentation and evaluation 33 months post-ART initiation.

On examination, he was alert but visibly anxious; temperature was normal, heart rate approximately 145 beats per minute, blood pressure 100/65 mmHg and respiratory rate 18/min. There was clear bilateral exophthalmos with lid retraction and lid lag, and a diffusely enlarged, smooth, soft, non-tender anterior neck mass that moved with deglutition. Cardiac auscultation revealed a tachycardic regular rhythm without murmurs; there were no signs of heart failure or focal neurologic deficits.There was no family history of thyroid disease or other autoimmune conditions.There was no known exposure to iodinated contrast or exogenous thyroid hormone.

Laboratory testing at presentation demonstrated biochemical thyrotoxicosis with an elevated free T4 (FT4 2.5 ng/dL) and a suppressed thyroid-stimulating hormone (TSH <0.005 µIU/mL). Thyroid autoantibodies (anti-TPO and TSH-receptor antibodies) were not available at our centre. Neck ultrasound showed diffuse enlargement with multinodular echotexture consistent with a diffusely hypervascular goitre. ECG showed sinus tachycardia without ischemic changes. HIV monitoring at the time confirmed continued virologic suppression and sustained CD4 recovery.

A clinical and biochemical diagnosis of Graves disease was made on the basis of symptomatic thyrotoxicosis, orbitopathy and diffuse goitre, and the timing in relation to marked immune restoration on ART was considered consistent with immune reconstitution inflammatory syndrome manifesting as autoimmune thyroid disease. He was commenced on carbimazole and propranolol while continuing ART without interruption. Over serial outpatient follow-up, his symptoms abated, heart rate normalised, and thyroid function tests improved (FT4 1.07 ng/dL, TSH 3.4 µIU/mL), permitting down-titration of carbimazole to 10 mg daily. He remains under joint endocrine and infectious-disease follow-up with sustained HIV virologic control and ongoing surveillance for relapse or need for definitive thyroid therapy.

## Discussion

Graves’ disease as IRIS in HIV is a rare phenomenon. Immune restoration can trigger autoimmune thyroiditis or Graves’ hyperthyroidism [3], but published reports are limited. In one cohort, Graves’ IRIS was observed as a distinctive late-onset event, often 2–3 years after ART [10]. In addition, this study noted that Graves’ IRIS was associated with a marked CD4⁺ gain and was uncommon [10]. The present case fits this pattern: after a nadir CD4 count of 23 cells/mm³ and robust immune recovery (CD4 >1800 cells/mm³), thyrotoxicosis developed 33 months into ART. The timeline parallels other reports (median ∼38 months) [2], reflecting the delayed phase of naïve T-cell repopulation. Only a few dozen cases of AIDS-related Graves’ IRIS have been published overall [12], [13], and pediatric occurrences are especially scarce [11], [14]. One recent pediatric case report noted its uniqueness [14], and our report adds to this scant literature.

Differential diagnosis of thyroid dysfunction in HIV/ART is broad. Clinicians must exclude primary thyroid disorders (e.g., Graves’ disease) and ART-related effects on the thyroid [4], [15]. By definition, IRIS requires evidence of ART-induced virologic/immunologic response followed by new inflammation, with alternative causes (such as drug toxicity or an untreated infection) excluded [1], [16]. IRIS typically occurs in patients with advanced HIV (very low CD4 counts) who then experience immune recovery on ART [16]. Distinguishing IRIS-associated Graves’ from coincident Graves’ disease depends chiefly on timing relative to ART and immune recovery [3], [4]. In this case, the onset of hyperthyroidism after ART initiation and rising CD4 count strongly favored an IRIS mechanism.

The diagnosis of Graves’ IRIS relies on standard criteria for Graves’ disease. In our patient, the combination of classic hyperthyroid symptoms (weight loss, palpitations, tremor) together with diffuse goiter and lid retraction strongly suggested Graves’ disease. Biochemical testing supported the diagnosis with suppressed TSH and elevated FT4. Thyroid ultrasound showed a diffusely enlarged, hypervascular gland, and the presence of bilateral exophthalmos confirmed the diagnosis in the absence of thyroid autoantibody assays [5]. Although testing for TSH-receptor antibodies is the most specific method for diagnosing Graves’ disease [7], it was not available at our center.This highlights an important diagnostic challenge: in resource-limited settings without access to antibody testing, characteristic orbital signs combined with ultrasound findings can support the diagnosis of Graves’ disease [5].

Management followed standard Graves’ therapy. We continued ART (since non-life-threatening IRIS generally does not require ART interruption [5]) and initiated symptomatic treatment with propranolol alongside antithyroid therapy. Methimazole (carbimazole) is first-line in children [5], [7]. The patient responded well: hyperthyroid symptoms abated and FT4 normalized, as seen in other series [2]. In published cases, antithyroid drugs and/or definitive therapy are effective. For example, a case series reported remission of hyperthyroidism in all treated patients (some after radioiodine ablation) [2]. Our patient’s improvement on carbimazole without relapse parallels these outcomes. Nonetheless, the relapse risk after stopping medication is unknown. Current practice (as in general Graves’ disease) is if hyperthyroidism recurs, definitive therapy (radioiodine or surgery) is recommended [5], [6]. Ongoing follow-up is needed, monitoring for thyroid function and reemergence of autoimmunity.

Prognosis in Graves’ IRIS appears favorable when treated [3], [12]. There are no reports of mortality attributable to Graves’ IRIS; the main risks are those of untreated hyperthyroidism and orbital disease. In adults, up to 25–30 % of ART-related Graves’ patients had some degree of Graves’ orbitopathy [17], but ours had only mild exophthalmos without optic involvement. His outcome (stable euthyroid on low-dose carbimazole) is similar to that of other reported cases [2]. This case reinforces that HIV-infected patients can have endocrine autoimmune manifestations post-ART, and that Graves’ disease should be considered if thyrotoxic signs emerge during immune recovery [3], [12], [14]. Given the frequent comorbidities in HIV, a high index of suspicion is warranted.

## Conclusion

Graves’ disease may rarely emerge as a late manifestation of immune reconstitution following effective ART, even in paediatric patients with vertically acquired HIV. Clinicians caring for children who demonstrate marked CD4 recovery should maintain a low threshold for evaluating unexplained weight loss, palpitations, heat intolerance, or ocular features, as these may reflect autoimmune thyroid disease rather than infection or ART toxicity. In resource-limited settings where antibody testing is unavailable, the combination of characteristic clinical features, biochemical thyrotoxicosis, and supportive ultrasound or orbital findings can justify initiating antithyroid therapy. Continuation of ART while treating hyperthyroidism with antithyroid drugs and symptomatic beta-blockade is generally effective and avoids interrupting immune recovery. Longitudinal endocrine follow-up is essential to monitor for relapse or progression and to plan definitive therapy if needed. Reporting such cases will increase awareness and help refine diagnostic and management strategies for IRIS-associated autoimmunity in pediatric HIV care.

## CRediT authorship contribution statement

**Biruk T. Mengistie:** Writing – review & editing, Writing – original draft. **Haile Asteway Mulat:** Writing – original draft, Supervision, Conceptualization. **Elezer B. Zewde:** Writing – review & editing, Data curation. **Chernet T. Mengistie:** Writing – original draft, Resources, Data curation. **Bezawit M. Haile:** Writing – original draft, Supervision, Conceptualization. **Addis H. Bekele:** Writing – review & editing, Visualization.

## Consent for publication

Written permission for publication of the clinical details and accompanying images was obtained from the patient’s legal guardian; the signed consent form is held by the corresponding author and can be made available to the Editor on request

## Ethical approval

IRB review and approval were waived for this case report.

## Funding

This research did not receive any specific grant from funding agencies in the public, commercial, or not-for-profit sectors.

## Declaration of Competing Interest

The authors declare that they have no known competing financial interests or personal relationships that could have appeared to influence the work reported in this paper.

## Data Availability

The data underlying the results presented in this work are available within the manuscript.
